# Role of brain-derived neurotrophic factor in the excitatory–inhibitory imbalance during the critical period of postnatal respiratory development in the rat

**DOI:** 10.14814/phy2.12631

**Published:** 2015-11-24

**Authors:** Xiu-ping Gao, Hanmeng Zhang, Margaret Wong-Riley

**Affiliations:** 1 Department of Cell Biology, Neurobiology and Anatomy, Medical College of WisconsinMilwaukee, Wisconsin

**Keywords:** Brain-derived neurotrophic factor, critical period, hypoglossal motoneurons, hypoxia, NTS_VL_, TrkB

## Abstract

The critical period of respiratory development in rats is a narrow window toward the end of the second postnatal week (P12–13), when abrupt neurochemical, electrophysiological, and ventilatory changes occur, when inhibition dominates over excitation, and when the animals’ response to hypoxia is the weakest. The goal of this study was to further test our hypothesis that a major mechanism underlying the synaptic imbalance during the critical period is a reduced expression of brain-derived neurotrophic factor (BDNF) and its TrkB receptors. Our aims were to determine (1) that the inhibitory dominance observed in hypoglossal motoneurons during the critical period was also demonstrable in a key respiratory chemosensor, NTS_VL_; (2) if in vivo application of a TrkB agonist, 7,8-DHF, would prevent, but a TrkB antagonist, ANA-12, would accentuate the synaptic imbalance; and (3) if hypoxia would also heighten the imbalance. Our results indicate that (1) the synaptic imbalance was evident in the NTS_VL_ during the critical period; (2) intraperitoneal injections of 7,8-DHF prevented the synaptic imbalance during the critical period, whereas ANA-12 in vivo accentuated such an imbalance; and (3) acute hypoxia induced the weakest response in both the amplitude and frequency of sEPSCs during the critical period, but it increased the frequency of sIPSCs during the critical period. Thus, our findings are consistent with and strengthen our hypothesis that BDNF and TrkB play a significant role in inducing a synaptic imbalance during the critical period of respiratory development in the rat.

## Introduction

Postnatal development of the rat’s respiratory system does not follow a straight, smooth path. Rather, abrupt but consistent changes occur in multiple brain stem respiratory-related nuclei during a critical period toward the end of the second postnatal week, when inhibitory synapses dominate over excitatory ones, when multiple neurochemical changes occur, and when the animals’ response to hypoxia is at its weakest (reviewed in Wong-Riley and Liu 2005, 2008; Liu et al. 2009; Gao et al. 2011). Excitatory neurochemicals such as glutamate and *N*-methyl-_D_-aspartate (NMDA) receptors exhibit a sudden fall in their expression on postnatal day (P) 12, whereas inhibitory ones such as GABA, GABA_B_ receptors, and glycine receptors are up-regulated (Liu and Wong-Riley 2002, 2005). GABA_A_ receptors undergo subunit switches, as do NMDA receptors around that time (Liu and Wong-Riley 2004, 2006, 2010c). Concomitantly, there is a switch in dominance from the chloride intruder Na^+^K^+^-2Cl^−^ cotransporter 1 (NKCC1) to that of the chloride extruder K^+^Cl^−^ cotransporter 2 (KCC2) (Liu and Wong-Riley 2012). Significantly, the expression of the serotonergic system, including the synthesizing enzyme tryptophan hydroxylase (TPH), the serotonin transporter SERT, and multiple serotonergic receptor subunits all fall precipitously at P12, followed by either a plateau or a rise close to that of the precritical period (Liu and Wong-Riley 2008, 2010a,b). At the synaptic level, both miniature and spontaneous excitatory postsynaptic currents (mEPSCs and sEPSCs) are significantly reduced at P12-13, whereas those of inhibitory ones (mIPSCs and sIPSCs) are significantly increased (Gao et al. 2011). At the system’s level, both the ventilatory and metabolic rate responses to acute hypoxia are significantly lower than those just before or after the critical period (Liu et al. 2006, 2009).

What could be the underlying mechanism for these changes during the critical period? Multiple factors are possible. However, the foremost one deserving serious consideration regarding synaptic imbalance is a known regulator of neuronal development and plasticity, that is, brain-derived neurotrophic factor (BDNF) and its high-affinity receptor, tropomyosin receptor kinase or tyrosine protein kinase B (TrkB) receptors (Barde et al. 1982; Klein et al. 1993). BDNF and TrkB are essential for the development of the respiratory system and for normal breathing behavior (Katz 2005). Brain-derived neurotrophic factor has also been reported to enhance excitation and suppress inhibition (Levine et al. 1995; Tanaka et al. 1997; Poo 2001). Specifically, BDNF increases the spontaneous firing rate of neurons as well as the amplitude and frequency of EPSCs (Levine et al. 1995), but it significantly reduces both evoked and spontaneous IPSCs as well as attenuated GABA_A_ receptor-mediated responses to exogenous GABA (Tanaka et al. 1997). Brain-derived neurotrophic factor is also known to increase presynaptic glutamate release (Schinder et al. 2000). Thus, if the level of BDNF is reduced during the critical period, then it may contribute to the heightened inhibition and reduced excitation at that time. Indeed, the expressions of both BDNF and TrkB are significantly reduced in multiple brain stem respiratory-related nuclei during the critical period (Liu and Wong-Riley 2013). Exogenous BDNF in vitro significantly increased sEPSCs and decreased sIPSCs in hypoglossal motoneurons (HMs) in brain stem slices during the critical period (Gao et al. 2014). The goal of this study was to test our hypothesis that the suppressed EPSCs during the critical period can be reversed by an in vivo administration of TrkB agonist (7,8-DHF; Jang et al. 2010) and that a TrkB antagonist (ANA-12; Cazorla et al. 2011) would accentuate such an imbalance during the critical period. We also wished to know if the imbalance observed in HMs can be documented in another key respiratory-related nucleus, specifically the ventrolateral subnucleus of the solitary tract nucleus (NTS_VL_). As a subset of NTS_VL_ neurons receives direct input from the carotid body, a known peripheral chemoreceptor sensitive to hypoxia (Finley and Katz 1992), and our previous study indicated a weaker ventilatory response to hypoxia during the critical period (Liu et al. 2006), we wished to test if hypoxia in vitro in the NTS_VL_ would induce a greater synaptic inhibition during the critical period.

## Materials and Methods

All animal procedures and experiments were performed in accordance with the Guide for the Care and Use of Laboratory Animals (National Institutes of Health Publications No. 80-23, revised 1996), and all protocols were approved by the Medical College of Wisconsin Animal Care and Use Committee (approval can be provided upon request). All efforts were made to minimize the number of animals used and their suffering.

A total of 169 Sprague–Dawley rats from 32 L were used. The animals were divided into six groups: (a) normal untreated controls for electrophysiological analyses of NTS_VL_ neuronal development at postnatal days P10-11 (before the critical period), P12-13 (during the critical period), and P14-15 (after the critical period); (b) intraperitoneally (i.p.) injected with 7,8-DHF (dissolved first in 100% DMSO, then diluted in 10% DMSO with 0.1 mol/L phosphate buffer; 5 mg/kg, once a day for 2 days and recorded on the third day before the critical period (P10-11), during the critical period (P12-13), and after the critical period (P14-15); the chosen dosage was based on published work (Jang et al. 2010); (c) littermates of b were i.p. injected with a comparable volume of vehicle (10% DMSO) once a day for 2 days and followed the same regimen as in b; (d) i.p. injected with ANA-12 (dissolved first in 100% DMSO, then diluted sequentially to 10% DMSO with 0.1 mol/L phosphate buffer; 2.46 mmol/kg, once a day at P7, P11, P12, or P15, and the HMs recorded a day after each injection); the chosen dosage was based on published report (Cazorla et al. 2011); (e) littermates of d were i.p. injected with a comparable volume of vehicle following the same regimen; and (f) electrophysiological study of NTS_VL_ neurons subjected to acute hypoxia (95% N_2_ and 5% CO_2_ for 5–6 min) in brain slices at P11 (before the critical period), P13 (during the critical period), and P15 (after the critical period). Hypoxia was 5 min for EPSC and 6 min for IPSC recordings because the peak of IPSCs lagged about 1 min behind that of EPSCs, so the extra min allowed for comparable elapse time after the peak response. The reason that recordings were done on NTS_VL_ for 7,8-DHF, but on HMs for ANA-12 is that we had already tested exogenous BDNF in vitro on HMs (Gao et al. 2014), but had not tested exogenous TrkB antagonist on HMs. Once we found out that neurons in the two nuclei responded similarly to TrkB agonist and antagonist, we did not deem it necessary to carry out a full-scale study of ANA-12 on NTS_VL_ neurons.

### Brain stem slice preparations

The day after the injections, experimental and control rats were anesthetized with isoflurane inhalation and decapitated. Brain stems were promptly removed and kept in ice-cold sucrose–cerebrospinal fluid (sucrose–CSF) gassed with carbogen (95% O_2_-5% CO_2_). Sucrose–CSF contained the following (in mmol/L): 220 sucrose, 2.5 KCl, 1.25 NaH_2_PO_4_, 0.5 CaCl_2_, 7 MgSO_4_, 26 NaHCO_3_, 25 glucose, 11.6 sodium ascorbate, and 3.1 sodium pyruvate, pH 7.4. Horizontal slices (300 mm thick) of the brain stem containing the hypoglossal nucleus or NTS_VL_ were cut with a Vibratome (Microslicer DTK-1000, Ted Pella, Inc., Redding, CA) and placed in ice-cold sucrose–CSF gassed with carbogen. The slices were transferred to an incubation chamber and maintained for 1 h in artificial CSF (ACSF) that contained the following (in mmol/L): 119 NaCl, 3 KCl, 2 CaCl_2_, 2 MgCl_2_, 1.25 NaH_2_PO_4_, 26 NaHCO_3_, and 10 glucose. The ACSF was saturated with carbogen (95% O_2_-5% CO_2_) at room temperature (22.5°C).

### Whole-cell patch-clamp recordings

Individual slices were transferred to a recording chamber on the microscope stage equipped with infrared-differential interference contrast microscopy (Olympus BX51W1, Olympus America Inc., PA, USA). Slices were submerged with a constant flow of oxygenated ACSF and stabilized with platinum wire weights. HMs and neurons in the NTS_VL_ were identified by their location, cell size, and shape (Liu and Wong-Riley 2005; Gao et al. 2011).

For recording sIPSCs, 10 *μ*mol/L 6-cyano-7-nitroquinoxaline-2,3-dione (CNQX; an AMPA/kainate receptor antagonist) and 25 *μ*mol/L D-2-amino-5-phosphonopentanoic acid (D-APV; a NMDA receptor antagonist) were added to the ACSF to block AMPA and NMDA currents, respectively. For recording sEPSCs, 1 *μ*mol/L strychnine (a glycine receptor antagonist) and 10 *μ*mol/L bicuculline (a GABA_A_ receptor antagonist) were added to ACSF to block glycinergic and GABAergic currents, respectively. For recording mIPSCs and mEPSCs, 0.5 *μ*mol/L tetrodotoxin (TTX; a voltage-dependent Na^+^ channel blocker) was also included in the ACSF.

Patch pipettes were pulled from borosilicate (KG-33) glass capillary tubings (1.5 mm outer diameter; 1.0 mm internal diameter; King Precision Glass, Claremont, CA) with a Narishige PC-10 two-stage electrode puller (Narishige International Inc., East Meadow, NY). When recording sIPSCs or mIPSCs, the pipette solution formulation was as follows (in mmol/L): 135 CsCl, 10 HEPES, 10 EGTA, 1.2 MgCl_2_, 2 MgATP, 0.3 Na_2_GTP, and 10 Na_2_-phosphocreatine, pH 7.25 (titrated with CsOH). When recording sEPSCs or mEPSCs, the pipette solution consisted of (in mmol/L): 125 Cs-gluconate, 10 CsCl, 10 HEPES, 10 EGTA, 1.2 MgCl_2_, 2 MgATP, 0.3 Na_2_GTP, and 10 Na_2_-phosphocreatine (pH = 7.25). The pipette resistance was 3–5 MΩ when filled with the above solution. Spontaneous and miniature IPSCs and EPSCs were recorded at a *V*_H_ of −70 mV in HMs. Whole-cell recordings were made using a patch-clamp amplifier (Multiclamp 700B; Molecular Devices, Union City, CA). Data acquisition and analysis were performed using a digitizer (DigiData 1440A) and analysis software pClamp 10 (Molecular Devices) as well as Mini Analysis v. 6.0.3 (Synaptosoft, Decatur, GA). Signals were filtered at 2 kHz and sampled at 10 kHz. Series resistance (10–25MΩ) was monitored throughout the recordings and data were discarded if the resistance changed by >20%. We performed series resistance compensation (70–80%) for sEPSCs and sIPSCs because their larger amplitude may introduce voltage-clamp errors. However, series resistance compensation was not done for miniature PSCs because the amplitude was very small (max 200 pA, mean 15–90 pA) and did not cause significant voltage-clamp errors (<6 mV under maximal 200 pA at 30 MΩ series resistance). We also wished to avoid increasing baseline noise. The basic electrophysiological characteristics of HMs and NTS_VL_, such as resting membrane potential, membrane capacitance, input resistance, rheobase current, and series resistance, were comparable to those described previously (Gao et al. 2011). All common chemicals were obtained from Sigma (St. Louis, MO). CNQX and all other drugs (e.g., antagonists) were from Tocris Bioscience (Ellisville, MO).

All recordings were performed at 32 ± 1°C using an automatic temperature controller (Warner Instrument, Hamden, CT). Recordings to be analyzed did not commence until responses were stabilized. We recorded for ∼10–30 min for each cell, and chose five continuous min of stabilized recording to analyze.

### Data analysis and statistics

**Figure 1:**
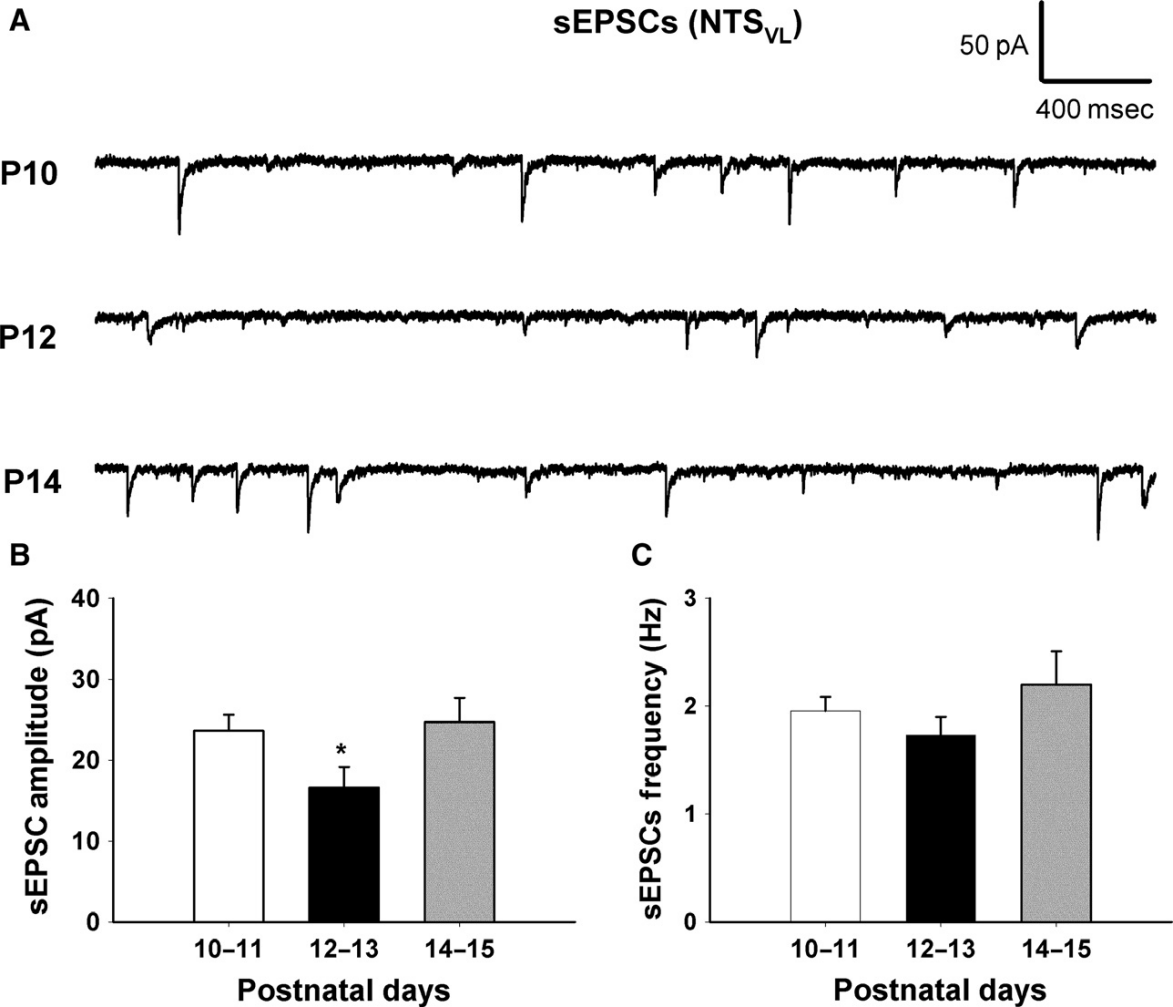
The amplitude of sEPSCs in normal NTS_VL_ neurons decreased during the critical period (P12-13). (A) Sample traces of sEPSCs recorded at different postnatal days (P10, P12 and P14). (B) Mean amplitude (in pA) of sEPSCs is shown at three time points (P10-11, P12-13, and P14-15). It exhibited a significant decrease at P12-13 (*P *<* *0.05). (C) Mean frequency (in Hz) of sEPSCs is displayed at three time point (P10-11, P12-13 and P14–15). Data are presented as mean ± SEM, *P <* *0.05 (significance between adjacent age groups).

**Figure 2:**
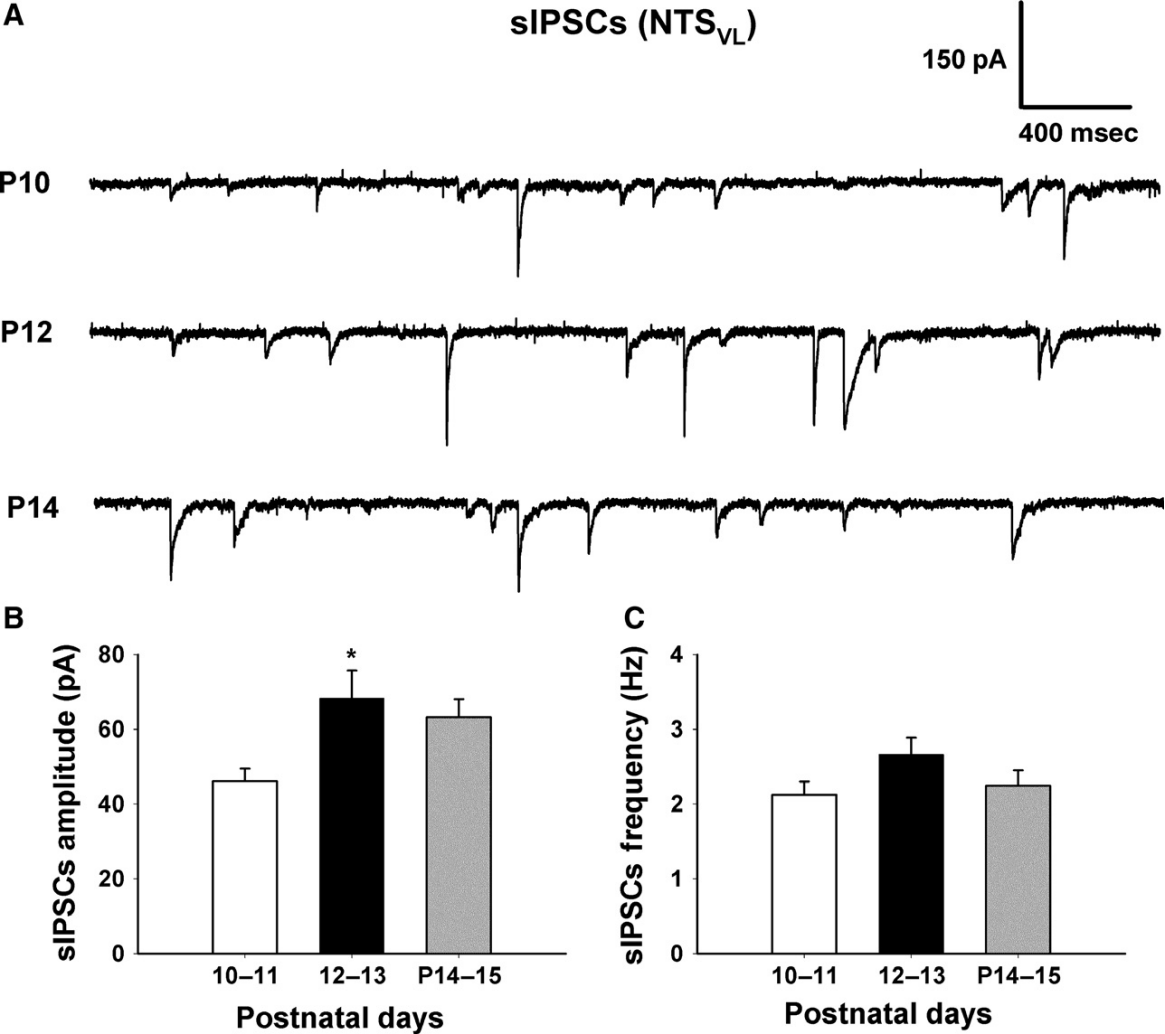
The amplitude of sIPSCs in normal NTS neurons increased during the critical period (P12–13). (A) Sample traces of sIPSCs recorded at different postnatal days (P10, P12, and P14). (B) Mean amplitude (in pA) of sIPSCs is shown at three time points (P10–11, P12–13, and P14–15). A significant increase is evident at P12–13 (*P *<* *0.05). (C) Mean frequency (in Hz) of sIPSCs is given at three time points (P10–11, P12–13, and P14–15). Data are presented as mean ± SEM, *P <* *0.05 (significance between adjacent age groups).

The amplitude and frequency of spontaneous and miniature EPSC and IPSC were determined by Minianalysis software (Mini Analysis software version 6, Synaptosoft). Effects of in vivo 7,8-DHF or ANA-12 on PSC amplitude and frequency were assessed for each cell. Data are presented as the mean ± SEM. One-way ANOVA was used to compare PSC responses at different ages for NTS_VL_ neurons (Figs.1 and 2). Two-way ANOVA was used for Figures6 for analysis of both drugs and ages. For comparisons between control and drug at each age group, Students’ *t*-test was performed followed by Bonferroni correction. For Figures7 and 8, the area under the curve was used for comparison followed by one-way ANOVA. Values of *P *<* *0.05 were considered significant.

**Figure 3:**
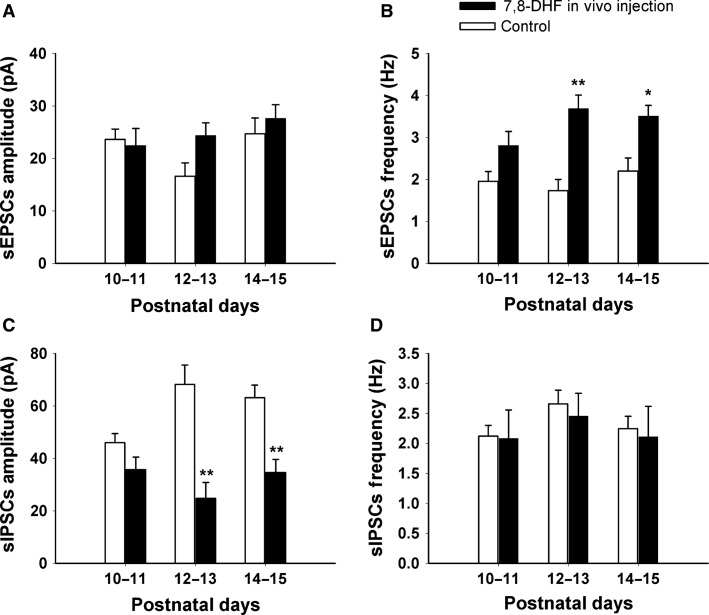
Amplitude and frequency of sEPSCs and sIPSCs in NTS_VL_ neurons after in vivo application of 7,8-DHF. (A) The amplitude of sEPSCs was increased by 7,8-DHF during the critical period, although it did not reach statistical significance. Changes in the amplitude before (P10–11) and after (P14–15) the critical period were also not significant. (B) The frequency of sEPSCs increased significantly with 7,8-DHF during (P12–13, *P *<* *0.01) and after (P14–15, *P *<* *0.05) the critical period as compared to respective controls. However, the frequency of sEPSC at P10–11 was not affected by the drug. (C) The amplitude of sIPSCs decreased significantly with 7,8-DHF at P12–13 (*P *<* *0.01) and P14–15 (*P *<* *0.01). However, the amplitude was not affected by 7,8-DHF at P10–11. (D) The frequency of sIPSCs recorded at different postnatal days (P10–11, P12–13, and P14–15) after 7,8-DHF was not significantly different from that of controls. Data are presented as mean ± SEM, *P <* *0.05, **P <* *0.01 (significance between control and 7,8-DHF-treated groups).

**Figure 4:**
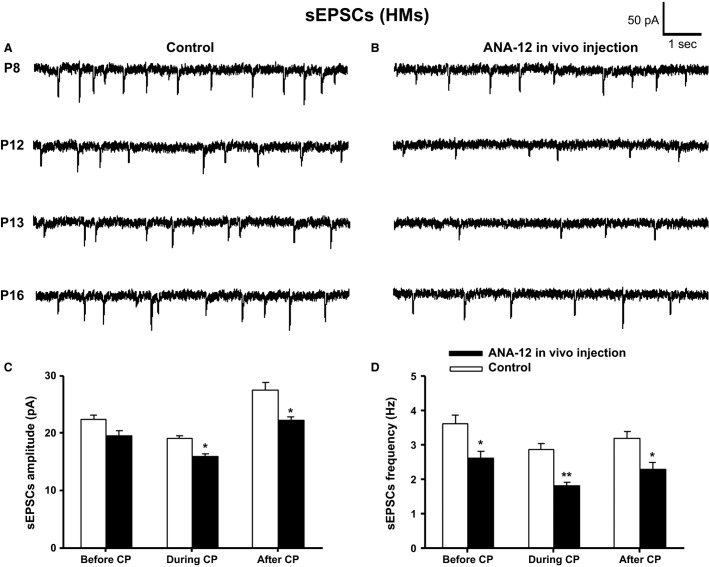
In vivo application of ANA-12 significantly decreased both the amplitude and frequency of sEPSCs in HMs. Sample traces of sEPSCs in controls (A) and ANA-12 injected (B) rats recorded at postnatal days P8, P12, P13, and P16, with a single i.p. injection of ANA-12 given 1 day before each recording. Mean amplitude (in pA) (C) and frequency (in Hz) (D) of sEPSCs at P8 (before the critical period CP), P12 and P13 (during the critical period), and P16 (after the critical period) of control and ANA-12 injected rats are shown. ANA-12 injections induced a significant decrease in the amplitude and frequency of sEPSCs during and after the critical period, as well as a significant decrease in frequency before the critical period (*P <* *0.05, **P <* *0.01).

**Figure 5:**
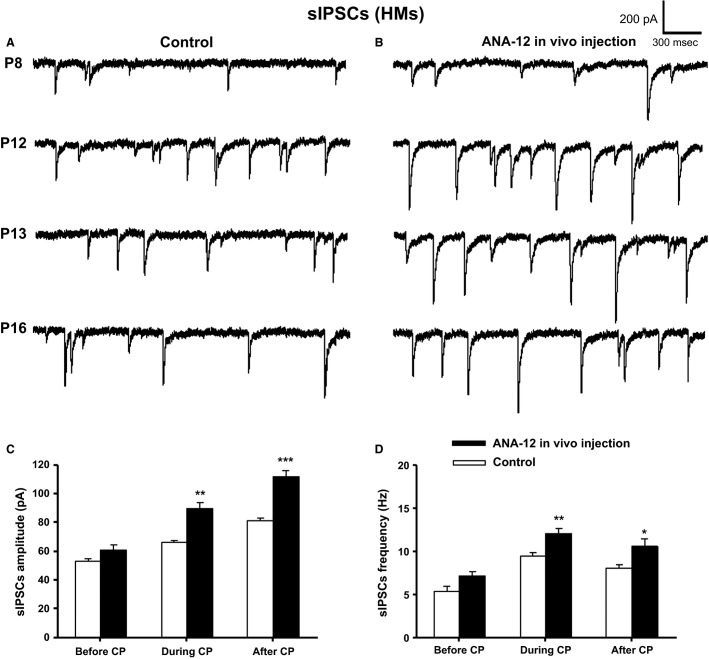
In vivo application of ANA-12 significantly increased both the amplitude and frequency of sIPSCs in HMs. Sample traces of sIPSCs in controls (A) and ANA-12 injected (B) rats recorded at postnatal days P8, P12, P13, and P16, with a single i.p. injection of ANA-12 given 1 day before each recording. Mean amplitude (in pA) (C) and frequency (in Hz) (D) of sIPSCs before, during, and after the critical period of control and ANA-12 injected rats are shown. ANA-12 injections induced a significant increase in amplitude and frequency of sIPSCs during and after the critical period (*P <* *0.05, **P <* *0.01, ***P <* *0.001), but not before the critical period.

**Figure 6:**
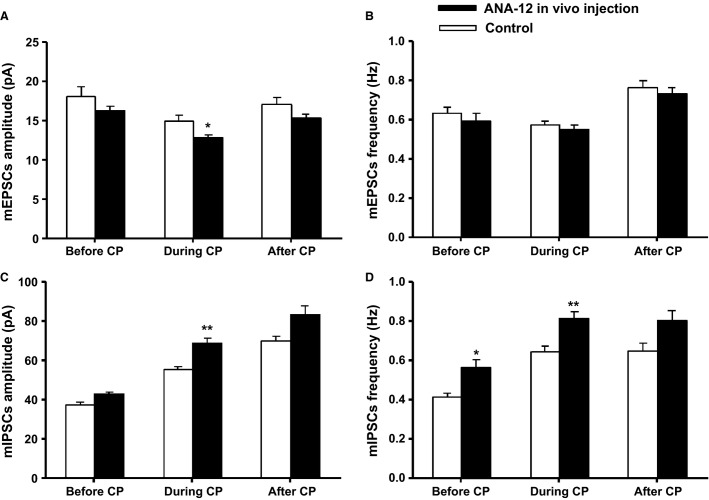
In vivo application of ANA-12 induced changes in mEPSCs and mIPSCs in HMs. Mean amplitude (in pA) (A) and frequency (in Hz) (B) of mEPSCs before, during, and after the critical period of control and ANA-12 injected rats are shown. Although all ages exhibited a decreasing trend in both amplitude and frequency of mEPSCs, only the amplitude at P12 had a significant decrease (*P <* *0.05). On the other hand, ANA-12 effectively increased both the amplitude (C) and frequency (D) of mIPSCs during the critical period, and increased the frequency before the critical period (*P <* *0.05, **P <* *0.01).

**Figure 7:**
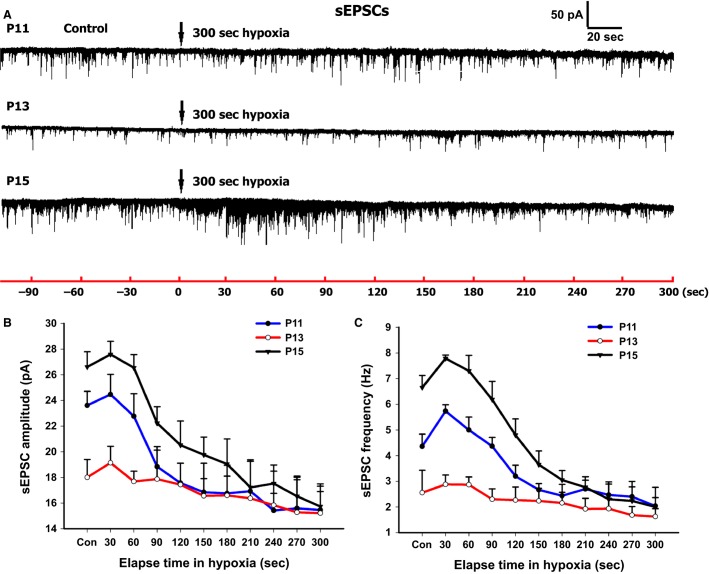
Effect of acute hypoxia on sEPSCs of NTS_VL_ neurons. (A) Representative traces of sEPSCs in NTS_VL_ neurons before and after acute hypoxia (95% N_2_ and 5% CO_2_ for 5 min) at P11, P13, and P15. (B) The amplitude of sEPSCs was lowest at P13 compared to the other two time points. Hypoxia induced the weakest response at P13. (C) The frequency of sEPSCs was lowest at P13 among the three time points. Hypoxia induced a biphasic response at P11 and P15, but not at P13.

**Figure 8:**
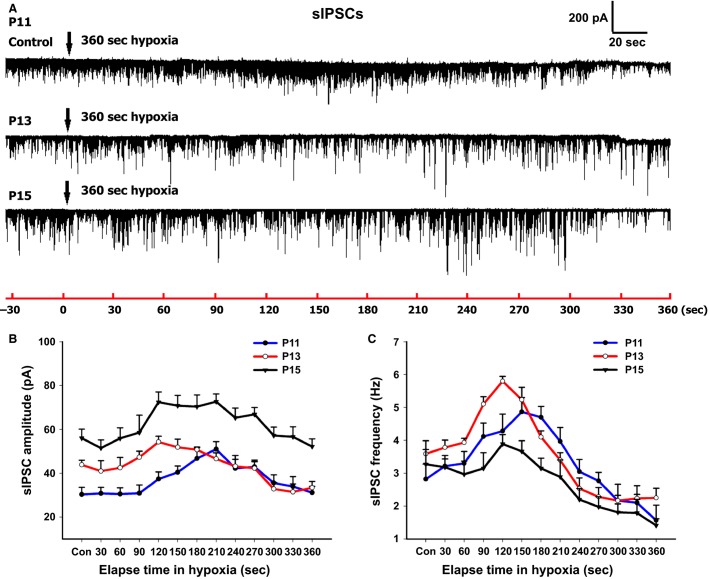
Effect of acute hypoxia on sIPSCs of NTS_VL_ neurons. (A) Representative traces of sIPSCs in NTS_VL_ neurons before and after acute hypoxia (95% N_2_ and 5% CO_2_ for 6 min) at P11, P13, and P15. (B) The amplitude of sIPSCs at P13 was in between those of the other two time points. Hypoxia induced an increase in the amplitude of sIPSCs above those of controls between 1.5 and 3 min after hypoxia at all three ages. The pattern of initial low followed by a rise in both the amplitude and frequency of sIPSCs in hypoxia was opposite to that of sEPSCs (compare with Fig.7). (C) Hypoxia induced a rise in the frequency of sIPSCs above control levels after 1.5–3 min of exposure at all three ages, but the increase at P13 was significantly above that of P15 (*P *<* *0.05).

## Results

### Developmental changes in the electrophysiological properties of NTS_VL_ neurons

NTS_VL_ neurons in brain stem slices were identified by their location (Liu and Wong-Riley 2005). Whole-cell patch-clamp recordings were made at a holding potential of −70 mV. Spontaneous EPSCs and IPSCs were recorded from these neurons at P10–11 (before the critical period), P12–13 (during the critical period), and P14–15 (after the critical period). As shown in Figure1, the amplitude of sEPSCs was significantly lower during the critical period (16.592 ± 2.537) than before (23.615 ± 1.960) or after (24.683 ± 3.014) that time (*P *<* *0.05; *n* = 10–11 for each group). The frequency of sEPSCs at P12-13 was only 1.731 ± 0.167; however, it did not reach statistical significance as compared to before (1.955 ± 0.132) or after (2.196 ± 0.311) that period. On the other hand, the amplitude of sIPSC was significantly higher during the critical period (68.227 ± 7.394) than before (46.021 ± 3.457) or after (63.166 ± 4.778) that time (*P *<* *0.05; *n* = 10 for each group) (Fig.2). The frequency of sIPSCs at P12–13 was 2.658 ± 0.228, but again, it was not statistically different from earlier (2.122 ± 0.176) or later (2.245 ± 0.206) periods (Fig.2).

### Effect of in vivo 7,8-DHF on sEPSCs

Rat pups were i.p. injected with 7,8-DHF once a day for 2 days and recorded on the third day either before the critical period (P10–11), during the critical period (P12–13), or after the critical period (P14–15). Whole-cell patch-clamp recordings of sEPSCs were done on NTS_VL_ neurons. Figures3A, B show that under control conditions, both the amplitude and frequency of sEPSCs had low values during the critical period (P12–13) as compared to before or after the critical period (see also Fig.1). However, in vivo 7,8-DHF increased the amplitude of sEPSCs by 46.67% above controls (control: 16.59 ± 2.5, *n* = 11; 7,8-DHF: 24.337 ± 2.412, *n* = 12) during the critical period, although it did not reach statistical significance. Likewise, there were no statistically significant changes in the amplitude of sEPSCs either before (control: 23.615 ± 1.96, *n* = 10; 7,8-DHF: 22.443 ± 3.254, *n* = 9) or after (control: 24.683 ± 3.014, *n* = 10; 7,8-DHF: 27.581 ± 2.635; *n* = 10) the critical period. On the other hand, 7,8-DHF significantly increased the frequency of sEPSCs, by 113.01% at P12-13 (*P *<* *0.01; control: 1.731 ± 0.167, *n* = 11; 7,8-DHF: 3.685 ± 0.324, *n* = 12), and by 59.69% at P14–15 (*P *<* *0.05; control: 2.196 ± 0.311, *n* = 10; 7,8-DHF: 3.508 ± 0.253, *n* = 10). At P10–11, 7,8-DHF increased the sEPSC frequency by 43.56% (control: 1.955 ± 0.132, *n* = 10; 7,8-DHF: 2.806 ± 0.334, *n* = 9), but it did not reach statistical significance. Two-way ANOVA indicated that 7,8-DHF significantly altered the frequency of the overall sEPSCs at the tested ages (*P *<* *0.001).

### Effect of in vivo 7,8-DHF on sIPSCs

Whole-cell patch-clamp recordings of sIPSCs were made on NTS_VL_ neurons. In vivo 7,8-DHF for 2 days had the opposite effect on sIPSCs as it did on sEPSCs. As shown in Figure3C, 7,8-DHF significantly reduced the normally high amplitude of sIPSCs during the critical period by 63.77% (*P *<* *0.01; control: 68.227 ± 7.394, *n* = 10; 7,8-DHF: 24.715 ± 6.099, *n* = 11) and after the critical period by 45.17% (*P *<* *0.01; control: 63.166 ± 4.778, *n* = 10; 7,8-DHF: 34.631 ± 4.985, *n* = 9). Two-way ANOVA indicated that DHF significantly changed the amplitude of sIPSCs at both P12–13 and P14–15. There was a statistically significant interaction between age and drug (*P *=* *0.004). However, 7,8-DHF did not cause a statistically significant change in the amplitude of sIPSCs before the critical period (control 46.021 ± 3.457 vs. 7,8-DHF 35.682 ± 4.816), presumably because endogenous BDNF was relatively high at that time. 7,8-DHF also did not alter significantly the frequency of sIPSCs at any of the three time points tested (Fig.3D).

### Effects of in vivo ANA-12 on sEPSCs

Whole-cell patch-clamp recordings of sEPSCs were made on HMs the day after a single i.p. injection of ANA-12. Examples of original sEPSC traces at P8 (before the critical period), P12 and P13 (during the critical period), and P16 (after the critical period) for controls are shown in Figure4A and for ANA-12 injected ones in Figure4B. Two-way ANOVA indicated no significant interaction between age and drug for sEPSC amplitude (*P *=* *0.273) or frequency (*P *=* *0.926), indicating that the effects are similar among the age groups. Student’s *t*-test with Bonferroni correction revealed a significant decrease in the amplitude (Fig.4C) and frequency (Fig.4D) of sEPSCs during and after the critical period, and a significant reduction in frequency before the critical period. When ANA-12 was given at P7, the amplitude and frequency of sEPSCs were decreased by 13.03% (*P *=* *0.069; control: 22.49 ± 0.67, *n* = 17; ANA-12: 19.56 ± 0.91, *n* = 12) and 27.89% (*P *<* *0.05; control: 3.63 ± 0.23, *n* = 17; ANA-12: 2.62 ± 0.21, *n* = 12), respectively, at P8. When ANA-12 was given at P11, the amplitude and frequency of sEPSCs were decreased by 17.32% (*P *<* *0.05; control: 18.59 ± 0.63, *n* = 14; ANA-12: 15.37 ± 0.64, *n* = 18) and 37.3% (*P *<* *0.001; control: 2.79 ± 0.24, *n* = 14; ANA-12: 1.75 ± 0.15, *n* = 18), respectively, at P12. When ANA-12 was given at P12, the amplitude and frequency of sEPSCs were decreased by 17.48% (*P *<* *0.01; control: 19.85 ± 0.70, *n* = 12; ANA-12: 16.38 ± 0.61, *n* = 23) and 36.74% (*P *<* *0.01; control: 2.94 ± 0.27, *n* = 12; ANA-12: 1.86 ± 0.17, *n* = 23), respectively, at P13. The values of P12 and P13 were combined in Figure4C, D, yielding a *P* value of <0.05 for amplitude and <0.01 for frequency during the critical period. When ANA-12 was given at P15, the amplitude and frequency of sEPSCs were decreased by 19.48% (*P *<* *0.05; control: 27.62 ± 1.27, *n* = 19; ANA-12: 22.24 ± 0.63, *n* = 12) and 28.38% (*P *<* *0.05; control: 3.19 ± 0.21, *n* = 19; ANA-12: 2.28 ± 0.20, *n* = 12), respectively, at P16.

### Effects of in vivo ANA-12 on sIPSCs

Whole-cell patch-clamp recordings of sIPSCs were made on HMs the day after a single i.p. injection of ANA-12. Figure5A illustrates original sIPSC traces at P8, P12, P13, and P16 for control, whereas Figure5B shows traces for ANA-12 injected ones. Two-way ANOVA indicated a significant interaction between age and drug for sIPSC amplitude at all ages tested, but no interaction was found for the frequency of sIPSC, suggesting that the effect on frequency was similar at the ages tested. Comparisons between control and ANA-12 groups at each time point using Bonferroni revealed that before the critical period (at P7), the amplitude (Fig.5C) and frequency (Fig.5D) of sIPSCs were not significantly changed at P8. However, when ANA-12 was given close to the critical period (P11), the amplitude and frequency of sIPSCs were significantly increased by 50% (*P *<* *0.01; control: 63.01 ± 1.51, *n* = 10; ANA-12: 94.51 ± 8.31, *n* = 9) and 17.74% (*P *<* *0.05; control: 9.89 ± 0.594, *n* = 10; ANA-12: 12.71 ± 0.98, *n* = 9), respectively, at P12. When ANA-12 was given during the critical period (P12), the amplitude and frequency of sIPSCs were also significantly increased by 20.85% (*P *<* *0.05; control: 70.36 ± 1.67, *n* = 8; ANA-12: 85.03 ± 4.64, *n* = 10) and 16.38% (*P *<* *0.05; control: 8.96 ± 0.34, *n* = 8; ANA-12: 11.41 ± 0.73, *n* = 10), respectively, at P13. The values of P12 and P13 were combined in Figures5C, D, yielding a *P* value of <0.01 for both amplitude and frequency during the critical period. When ANA-12 was given after the critical period (P15), the amplitude and frequency of sIPSCs were likewise significantly increased by 37.72% (*P *<* *0.001; control: 81.18 ± 1.96, *n* = 23; ANA-12: 111.8 ± 4.48, *n* = 15) and 18.04% (*P *<* *0.01; control: 8.08 ± 0.35, *n* = 23; ANA-12: 10.62 ± 0.83, *n* = 15), respectively, at P16.

### Effects of in vivo ANA-12 on miniature PSCs

Miniature EPSCs and IPSCs were recorded from P8, P12, P13, and P16 HMs. ANA-12 did not significantly alter the amplitude or frequency of mEPSCs except during the critical period (Fig.6A, B), at which time the amplitude of mEPSCs was decreased by 19.19% (*P *<* *0.05). On the other hand, ANA-12 significantly increased the amplitude and frequency of mIPSCs during the critical period (*P *<* *0.01 for both) (Fig.6C, D). When recorded before the critical period, the amplitude and frequency of mIPSCs were increased by 13.68% (*P *>* *0.05) and 36.87% (*P *<* *0.05), respectively. When recorded during the critical period, the amplitude and frequency were increased by 26.74% (*P *<* *0.01) and 34.2% (*P *<* *0.01), respectively, at P12; and by 21.68% (*P *<* *0.01) and 20.65% (*P *<* *0.05), respectively, at P13. The values of P12 and P13 were combined in Figures6C, D, yielding a *P* value of <0.01 for both amplitude and frequency during the critical period. When recorded after the critical period, the amplitude and frequency were also increased by 19.28 and 23.49%, respectively, but they did not reach statistical significance.

### Response of NTS_VL_ neurons to hypoxia in brain slices

To determine if the cellular response to hypoxia was different during the critical period as opposed to other times, we subjected brain stem slices to 95% N_2_ and 5% CO_2_ for 5–6 min and performed whole-cell patch-clamp recordings of sEPSCs and sIPSCs on NTS_VL_ neurons. Figure7A shows representative traces of sEPSCs before and after hypoxia for 5 min at P11 (before the critical period), P13 (during the critical period), and P15 (after the critical period). In plotting the amplitude (Fig.7B) and frequency (Fig.7C) at control levels and every 30 sec during 5 min of hypoxia, it is clear that the values were the lowest during the critical period (P13), and hypoxia did not induce any rise in sEPSCs. On the other hand, the baseline values of sEPSC amplitude and frequency before and after the critical period (P11 and P15, respectively) were much higher than those at P13. Hypoxia at those times induced relatively high amplitude and frequency levels during the first 0.5 to 1 min followed by a fall to a much lower level for the rest of the 5 min exposure. Such a trend at the single-cell level is reminiscent of the biphasic response at the whole-animal level prominent in developing animals (Mortola 1984), but the response was weakest at P13 (Liu et al. 2006). Using the area under the plot curve followed by one-way ANOVA, we did not find any significant difference in the amplitude of sEPSCs among the three groups (P11: 5540.6 ± 505.59, *n* = 6; P13: 5083.54 ± 197.45, *n* = 8; P15: 6241.15 ± 529.8, *n* = 7). However, the frequency of sEPSCs at P13 was significantly lower than those at P15 (*P *<* *0.001) or at P11 (*P *<* *0.01), and the frequency at P11 was also significantly different from that at P15 (*P *<* *0.05) (P11: 1029.1 ± 82.10, *n* = 6; P13: 673.37 ± 56.77, *n* = 8; P15: 1333.99 ± 65.42, *n* = 7). When we compared the values at each of the time points among the three age groups, we found that there were significant differences in the amplitude between P13 and P15 at control, 30 sec, and 60 sec in hypoxia (*P *<* *0.05–0.01). Moreover, there were significant differences in the frequency of sEPSCs among the three groups at control, 30, 60, and 90 sec in hypoxia (*P *<* *0.05–0.001). Differences in frequency were also noted between P13 and P15, as well as between P11 and P15 at 120 and 150 sec in hypoxia (*P *<* *0.05–0.001). A significant difference in frequency remained between P13 and P15 at 180 sec in hypoxia (*P *<* *0.05).

With regard to sIPSCs, Figure8A shows representative traces at the same three time points before and after hypoxia for 6 min. The baseline (control) level of amplitude at P13 was in between those before and after the critical period (Fig.8B), whereas the frequency at P13 was not statistically different from the other time points (Fig.8C). Hypoxia, however, induced a dip in amplitude of sIPSCs in the initial 60 sec followed by a rise and a plateau for the next 3 min, a pattern that was exactly opposite that of sEPSC (see Fig.7). Hypoxia also induced a significant rise in the frequency of sIPSCs at P13 that was much greater than those at the other two time points. By comparing the areas under the plot curve followed by one-way ANOVA, a statistically significant difference was found among the treatment groups for both amplitude (*P *<* *0.001) and frequency *(P *<* *0.05). The values of amplitude at P11 and P13 were found to be significantly lower than that at P15 (P11 vs. P15, *P *<* *0.001; P13 vs. p15, *P *<* *0.01; P11: 13531.86 ± 1177.59, *n* = 5; P13: 15668.72 ± 1024.13, *n* = 9; P15: 22525.35 ± 1491.58, *n* = 6) (Fig.8B). However, the frequency at P13 was significantly higher than that at P15 (*P *<* *0.05; P11: 1219.08 ± 115.77, *n* = 5; P13: 1291.77 ± 60.10, *n* = 9; P15: 979.87 ± 92.95, *n* = 6) (Fig.8C). Both the amplitude and frequency of sIPSCs showed an increase above control levels between 1.5 and 3 min after hypoxia at all three time points tested. When we compared the values at each of the time points among the three age groups, we found that there were significant differences in the amplitude between P13 and P11 (*P *<* *0.05) and between P11 and P15 (*P *<* *0.001) at control levels. A significant difference in amplitude was also found between P13 and P15 at 60 and 120–360 sec, between P13 and P11 at 90 and 120 sec in hypoxia, and between P11 and P15 during the entire hypoxic period (*P *<* *0.05–0.001). Moreover, there were significant differences in the frequency of sIPSCs between P13 and P15 at 90–150 sec in hypoxia, and between P13 and P11 at 120 sec in hypoxia (*P *<* *0.05–0.001).

## Discussion

Our results indicate that (a) the synaptic imbalance that was found in hypoglossal motoneurons during the critical period was also evident in NTS_VL_; (b) in vivo administration of a TrkB agonist, 7,8-DHF, prevented the synaptic imbalance during the critical period; (c) a single i.p. injection of a TrkB antagonist, ANA-12, significantly enhanced inhibition and suppressed excitation especially during and after the critical period; and (d) acute hypoxia heightened the synaptic imbalance during the critical period. These findings are consistent with our hypothesis that reduced expressions of BDNF and TrkB contribute significantly to the synaptic imbalance within respiratory-related nuclei during the critical period of respiratory development in the rat, and that hypoxia enhances such an imbalance.

Brain-derived neurotrophic factor is the second neurotrophic factor purified after the discovery of nerve growth factor (Barde et al. 1982). It is widely expressed in the developing and mature CNS (Maisonpierre et al. 1990; Conner et al. 1997). BDNF and its high-affinity TrkB receptors play important roles in neuronal cell survival, differentiation, cell migration, neurite outgrowth, synapse formation, stabilization, and plasticity (Hofer and Barde 1988; Wardle and Poo 2003; Baker-Herman et al. 2004; Wilkerson and Mitchell 2009; Yoshii and Constantine-Paton 2010). In the respiratory system, BDNF is the only known neurotrophin found to be essential for the development of normal respiratory rhythm and ventilatory control. BDNF knockout mice exhibit severe respiratory abnormalities similar to those of sudden infant death syndrome (SIDS) and congenital central hypoventilation syndrome (CCHS), such as impaired hypoxic ventilatory response, abnormal respiratory pattern, periodic breathing, apnea, and abnormal fluctuations in respiratory patterns (Erickson et al. 1996; Balkowiec and Katz 1998). These mice die within 1–2 weeks of birth, most likely from breathing complications (Erickson et al. 1996). Knocking out BDNF’s high-affinity TrkB receptors also leads to early postnatal death (Klein et al. 1993). In the adult rat, both BDNF and TrkB are found in many regions of the brain, including many brain stem respiratory-related nuclei (Conner et al. 1997; Katz 2005; Tang et al. 2010; Liu and Wong-Riley 2013).

The widespread influence of BDNF over multiple developmental processes and especially over respiratory development suggests that any changes in its own expression will have overriding consequences. Indeed, the down-regulation of both BDNF and TrkB in multiple brainstem respiratory-related nuclei during the critical period (Liu and Wong-Riley 2013) coincides temporally with many major changes in the respiratory system. First, BDNF is known to enhance excitation and suppress inhibition (Kang and Schuman 1995; Levine et al. 1995; Tanaka et al. 1997; Brunig et al. 2001; Poo 2001; Henneberger et al. 2005). If the level of BDNF itself were reduced, it should result in reduced excitation and enhanced inhibition during the critical period, and that was precisely what was observed (Liu and Wong-Riley 2002, 2005; Gao et al. 2011). Second, BDNF is known to stimulate serotonin synthesis (Eaton et al. 1995; Siuciak et al.^1998^), and its own synthesis can be serotonin-dependent (Baker-Herman et al. 2004; Homberg et al. 2014). During the critical period, the expressions of many serotonergic neurochemicals that are known to modulate respiration and autonomic functions, including its synthesizing enzyme tryptophan hydroxylase, its transporter SERT, and its receptor subunits 5-HT 1A, 1B, and 2A, all fall abruptly at P12 in multiple brain stem respiratory-related nuclei (Liu and Wong-Riley 2008, 2010a,b). The temporal coincidence of such a fall with that of BDNF underscores their reciprocal relationship. Third, BDNF reportedly suppresses the expression of neuron-specific Cl^−^ exporter, KCC2, known to enhance fast GABAergic inhibition (Rivera et al.^2002^; Wardle and Poo 2003). Reduced BDNF expression during the critical period coincides temporally with a rise in the expression of KCC2 in multiple brain stem respiratory-related nuclei (Liu and Wong-Riley 2012) as well as a rise in inhibition (Liu and Wong-Riley 2002; Gao et al. 2011). These system-wide changes cannot be explained away as mere coincidence, especially when they occur so transiently over a 1- to 2-day period during normal postnatal respiratory development.

To rule out the possibility of a mere coincidence and to further test our hypothesis that BDNF/TrkB play a major role in the synaptic imbalance during the critical period, we previously tested with exogenous BDNF in vitro (Gao et al. 2014). We found that it significantly increased the normally lowered frequency of sEPSCs, but decreased the normally heightened amplitude and frequency of sIPSCs in hypoglossal motoneurons during the critical period. Exogenous BDNF also decreased the normally heightened frequency of mIPSCs at this time. The effect was partially blocked by a TrkB receptor antagonist, K252a. The current study extended the testing to an in vivo administration of a TrkB agonist, 7,8-DHF, and a TrkB antagonist, ANA-12. 7,8-DHF is a flavone derivative of low molecular weight that can easily cross the blood–brain barrier; it is also a bioactive high-affinity TrkB agonist that induces receptor dimerization and autophosphorylation as well as activation of downstream signaling (Jang et al. 2010). We found that it prevented the synaptic imbalance during the critical period by up-regulating excitation and reducing inhibition (present study). On the other hand, ANA-12 is a low-molecular weight TrkB ligand that also readily crosses the blood–brain barrier; it prevents TrkB receptor activation with a high potency and inhibits downstream signaling without altering TrkA or TrkC functions (Cazorla et al. 2011). We found that a single intraperitoneal injection of ANA-12 induced a significant rise in the amplitude and frequency of sIPSCs, but a fall in those of sEPSCs, especially during the critical period. It also increased the amplitude and frequency of mIPSCs, but decreased the amplitude of mEPSCs during the critical period (this study). These findings are consistent with and strengthen our hypothesis that a transient down-regulation of BDNF and TrkB during the critical period contributes to the synaptic imbalance at that time. Such imbalance is evident at the neurochemical level in many brain stem respiratory-related nuclei (Liu and Wong-Riley 2002, 2005). It is also evident at the electrophysiological level in not only the hypoglossal motoneurons (Gao et al. 2011), which are known to control tongue extruder muscles for upper airway patency (Lowe 1980), but also in the NTS_VL_ (this study), at least a subset of neurons of which are known to receive direct input from peripheral chemoreceptors, the carotid bodies (Finley and Katz 1992; Wong-Riley et al. 2013). Moreover, we found that the NTS_VL_ neurons responded to acute hypoxia with a much lower amplitude and frequency of sEPSCs, but a much higher frequency of sIPSCs during the critical period than either before or after the critical period. This finding at the synaptic level is consistent with what we had observed at the whole-animal pulmonary ventilation level that the response to acute hypoxia is the weakest during the critical period (Liu et al. 2006).

What can be the reason for the down-regulation of BDNF/TrkB during the critical period? We propose several factors: (1) Our electrophysiological data indicate that excitatory synapses develop and peak earlier than inhibitory ones, at least in hypoglossal motoneurons (Gao et al. 2011). This is consistent with our ultrastructural findings in the developing cerebellum (Mjaatvedt and Wong-Riley 1988). The expression of BDNF/TrkB is high during the period of excitatory synaptic growth and development (i.e., before the critical period) (Liu and Wong-Riley 2013), consistent with their role in enhancing excitatory synapses and in the formation of appropriate synaptic connections (Cohen-Cory et al. 2010). However, once the excitatory innervation has peaked, refinement of respiratory circuit requires inhibitory modulation to achieve finely controlled respiratory functions. Accomplishing this developmental transition necessitates a transient down-regulation (but not elimination) of BDNF/TrkB. Such down-regulation reduces excitation, but releases inhibition from BDNF-induced inhibition (disinhibition), allowing inhibitory synapses to flourish. Inhibition is further enhanced by the release of KCC2 from the suppressive action of BDNF. Heightened inhibition would suppress neuronal activity, leading to further reduction in activity-dependent BDNF/TrkB expression. Our findings thus far are all consistent with these scenarios.

Thus, the critical period of respiratory development is a transitional period between immaturity and maturity, that is, it is a natural process of *normal* development, and the down-regulation of BDNF/TrkB is a *natural* part of this process. However, this will create a window of vulnerability. When challenged with respiratory stressors such as acute hypoxia, the animals are not able to respond adequately during this time (Liu et al. 2006, 2009; this study). When challenged with more severe stressors, such as sustained hypoxia, the response may be absent and even death may ensue (Mayer et al. 2014). Unexpected deaths were also observed in 12-day-old rat pups given a sublethal dose of endotoxin 2 days after exposure to a nonlethal influenza a virus (Blood-Siegfried et al. 2002). Younger or older pups did not die from the same treatment. This has significant implication for SIDS, as presumably vulnerable infants die during a critical period of postnatal development when exposed to an external stressor, such as hypoxia (Filiano and Kinney 1994; Moon et al. 2007).

The critical period, however, is transitory, and, in time, a balanced state of excitation and inhibition is achieved (driven by presumed genetic programming and environmental influences). In mature neurons, BDNF/TrkB functions at a basal level to maintain and modulate synaptic activity and synaptic plasticity (Poo 2001; Yoshii and Constantine-Paton 2010).

## Conclusions

Our previous studies have established the neurochemical, ventilatory, and electrophysiological basis of a critical period of respiratory development in the rat. During this period, there is a marked synaptic imbalance between heightened inhibition and suppressed excitation. We hypothesized that the underlying mechanism is a transient, depressed expression of BDNF and TrkB receptors. This study documented that in vivo administration of a TrkB agonist, 7,8-DHF prevented, whereas a TrkB antagonist, ANA-12, accentuated the synaptic imbalance during the critical period, and that hypoxia also heightened such an imbalance. These findings are consistent with our hypothesis and strengthen the role of BDNF/TrkB in contributing to a synaptic imbalance during respiratory development.
