# Corrigendum

**DOI:** 10.14814/phy2.12842

**Published:** 2016-06-14

In Gao et al. 2015, the following error was published in the Materials and Methods section:

In paragraph 2, letter (d) indicated that the animals were given the dosage “ … 2.46 mmol/kg once a day at P7, P11 …. This should be corrected to “2.46 x 10^−3^ mmol/kg.” The correct dosage was given to animals.

The authors apologise for the typographical error.
